# Plasmonic Au–MoS_2_ Nanohybrids Using Pulsed Laser-Induced Photolysis Synthesis for Enhanced Visible-Light Photocatalytic Dye Degradation

**DOI:** 10.1021/acsomega.4c04687

**Published:** 2024-09-04

**Authors:** Yung-Mei Lin, Chieh-Ming Wu, Yi-Hsuan Lin, Jui-Hao Chang, Ling-Yi Liang, Vincent K. S. Hsiao, Chih-Chien Chu

**Affiliations:** † Department of Applied Materials and Optoelectronic Engineering, National Chi Nan University, Nantou 545301, Taiwan; ‡ Department of Medical Applied Chemistry, Chung Shan Medical University, Taichung 40201, Taiwan; § Department of Medical Education, Chung Shan Medical University Hospital, Taichung 40201, Taiwan

## Abstract

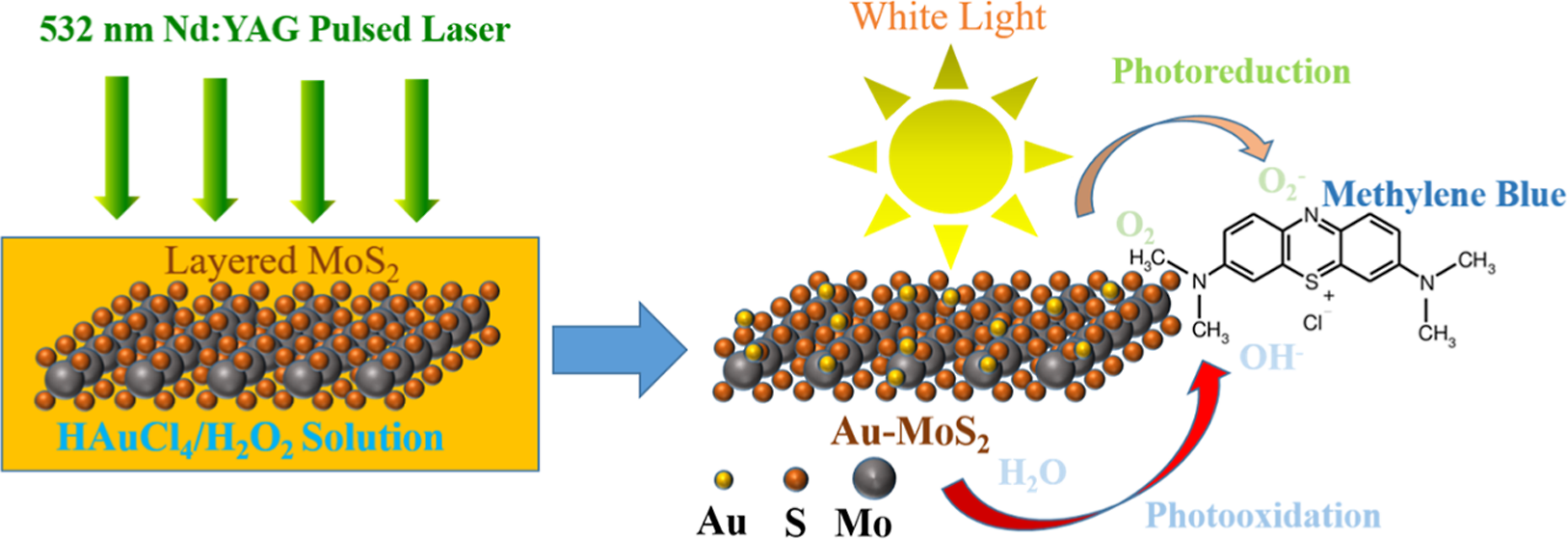

The Au–MoS_2_ nanocomposites (NCPs) exhibit excellent visible-light photocatalytic activity and potential applications in the photocatalytic degradation of organic dyes. In this study, an Au–MoS_2_ heterojunction structure with Au nanoparticles (NPs) deposited on MoS_2_ nanosheets was synthesized via the pulsed laser-induced photolysis method. The influence of Au content on the photocatalytic performance was systematically investigated, and the working mechanism under visible light excitation was elucidated. The optimal Au–MoS_2_ NCPs exhibited efficient degradation of methylene blue (MB) dye, mainly attributed to the plasmon resonance effect of Au NPs which facilitated the visible light harvesting and hot electron injection. The Au/MoS_2_ interface promoted the separation and transfer of photogenerated charge carriers. The electrostatic adsorption between positively charged MB molecules and the negatively charged MoS_2_ surface favored the affinity toward active sites. Furthermore, the photogenerated electrons and holes participated in generating reactive oxygen species such as superoxide and hydroxyl radicals, which initiated the oxidative degradation of MB. The PLIP-introduced Au NPs not only endowed the material with excellent visible light responsivity but also possibly modulated the electronic structure and photocatalytic active sites of MoS_2_ through an intrinsic effect, providing new insights for further enhancing the photocatalytic performance of Au–MoS_2_ NCPs.

## 1 Introduction

Photocatalysis plays a crucial role in environmental remediation and energy conversion. In the environmental domain, photocatalysis effectively degrades organic pollutants, heavy metal ions, and hazardous substances into harmless products,^1−3^ improving water and air quality and mitigating pollution’s adverse impacts. In the energy sector, photocatalysis efficiently converts solar energy into chemical energy, such as hydrogen production via water splitting,^4−6^ promoting clean energy generation and utilization. Additionally, photocatalysis can enhance resource utilization by converting wastewater pollutants into renewable resources. Developing visible-light-responsive photocatalysts is both necessary and challenging.^7−9^ The visible light region comprises a significant portion of the solar spectrum, and photocatalysts capable of absorbing and utilizing visible light can fully exploit solar resources, improving photocatalytic efficiency and reducing energy consumption and environmental impact. However, traditional photocatalytic materials, such as TiO_2_, exhibit limited absorption and utilization capabilities in this region due to visible light’s relatively low energy.^10^ Therefore, developing emerging materials to enhance visible light absorption and conversion efficiency is crucial, alongside addressing technical challenges like reaction condition optimization, catalyst stability, and activity.

TiO_2_ has been a research priority due to its exceptional photocatalytic performance.^11^ However, with a bandgap of approximately 3.2 eV, TiO_2_ can only respond to ultraviolet light below 400 nm, resulting in low visible light utilization efficiency and weak photocatalytic reduction capability for hydrogen production. To address these limitations, researchers have developed non-TiO_2_ photocatalytic materials, such as ZnO, Fe_2_O_3_, CdS, and ZnS.^12−15^ Among them, MoS_2_ has garnered significant attention due to its large surface area, open nanostructure, and adjustable interlayer spacing.^16−19^ As a layered semiconductor with a suitable bandgap in the visible-light region, MoS_2_ exhibits tremendous potential in visible-light photocatalysis and organic pollutant degradation. However, MoS_2_ still suffers from limited visible-light utilization and high recombination rates of photogenerated electron–hole pairs, restricting its large-scale applications, such as photocatalytic water splitting and organic pollutant degradation. Therefore, developing new photocatalytic materials with high visible light responsiveness and efficient charge carrier separation is crucial for improving overall catalytic efficiency and reducing energy consumption.

MoS_2_ is a typical two-dimensional (2D) layered semiconductor belonging to the transition metal dichalcogenide family.^20^ Its unique properties have garnered significant attention in optoelectronics, energy storage, biosensing, nanoelectronics, and photocatalysis. The tunable bandgap structure of MoS_2_ can be continuously adjusted from 1.9 eV (direct bandgap) to 1.2 eV (indirect bandgap) depending on the number of layers,^21^ enabling efficient absorption and utilization of visible and infrared light. The conduction band (CB) and valence band (VB) positions of MoS_2_, approximately −0.2 and +1.8 eV (vs NHE),^22^ suggest sufficient reduction and oxidation potentials to form superoxide and hydroxyl radicals during photocatalysis. Strategies to enhance photocatalytic activity of MoS_2_ include controlling its morphology and structure,^23^ doping,^24^ forming heterojunctions,^25^ modifying with carbon nanomaterials,^26^ and compositing with noble metal nanoparticles.^27,28^ The photocatalytic process of MoS_2_ involves the generation and migration of electron–hole pairs upon irradiation. The photogenerated electrons and holes participate in reduction and oxidation reactions, respectively, with adsorbed species on the MoS_2_ surface, generating reactive oxygen species (ROS) such as O_2_^–^ and OH radicals.

Recent studies have highlighted the potential of metal-loaded semiconductor photocatalysts,^29−37^ particularly Au nanoparticles (NPs) composited with MoS_2_, for enhanced visible light photocatalysis. These Au–MoS_2_ nanocomposites (NCPs) have found applications in organic dye degradation, electrochemical sensing, hydrogen evolution, carbon dioxide reduction, and water splitting.^38−42^ The synergistic effects of MoS_2_’s optical and electronic properties, Au NPs’ localized surface plasmon resonance (LSPR), and the formation of Schottky junctions at the Au/MoS_2_ interface contribute to the enhanced performance of these NCPs. Au–MoS_2_ is a heterogeneous NCP material formed by the integration of Au NPs with MoS_2_ nanosheets, achieving a synergistic combination of the advantages of both materials. Optically, MoS_2_ possesses a narrow bandgap, enabling efficient visible light absorption, while Au NPs enhance light absorption and localized electric fields due to the LSPR effect, providing the Au–MoS_2_ NCPs with exceptional visible light absorption and photoelectric conversion capabilities.

Compared to traditional photocatalytic materials (such as TiO_2_ and CdS), Au–MoS_2_ exhibits superior photocatalytic activity, primarily arising from the intrinsic excellent optical and electronic properties of MoS_2_, the LSPR surface effect of Au NPs, and the formation of Schottky junctions at the Au/MoS_2_ interface, favoring charge separation and transfer. Additionally, MoS2’s relatively high chemical stability and the protective effect of Au NPs modification render the Au–MoS_2_ composite more stable during photocatalytic reactions. Au–MoS_2_ NCPs have been employed for the efficient and eco-friendly degradation of various organic dye molecules.^35−37^ The remarkable photocatalytic activity of Au–MoS_2_ NCPs can be attributed to the large surface area of MoS_2_ nanosheets, facilitating efficient loading of Au NPs, and the unique metal–semiconductor interface effect, significantly enhancing the separation and transfer efficiency of photoinduced charges.

In this study, we successfully prepared visible light-driven Au–MoS_2_ NCP photocatalysts using the pulsed-laser-induced photolysis [pulsed laser-induced photolysis (PLIP)] technique and evaluated their visible-light photocatalytic degradation performance toward methylene blue (MB) dye. Compared to conventional synthesis techniques, the PLIP approach offers several distinct advantages. First, it enables the rapid, one-step formation of Au NPs directly on the MoS_2_ surface, ensuring intimate contact and strong coupling between the two components. Second, the PLIP process allows precise control over the size, distribution, and loading of Au NPs by tuning the laser parameters and precursor concentrations. This level of control is crucial for optimizing the photocatalytic performance of the resulting Au–MoS_2_ hybrids. Furthermore, the PLIP method is a green and efficient synthesis route, as it does not require harsh chemicals, high temperatures, or prolonged reaction times. These advantages make the PLIP technique a promising strategy for fabricating high-quality Au–MoS_2_ photocatalysts with tailored properties. Through material characterization techniques such as scanning electron microscopy (SEM), X-ray diffraction (XRD), and transmission electron microscopy (TEM), we confirmed the tight integration of Au NPs on the surface of the layered MoS_2_ substrate, forming an ideal heterogeneous nanostructure. The experimental results demonstrate that after modification by the PLIP process, the Au NPs grown on the surface of MoS_2_ significantly enhance its light absorption and photocatalytic activity in the visible light region. Electrochemical cyclic voltammetry (CV) studies revealed that increasing the concentration of the auric acid (HAuCl_4_) precursor effectively increases the loading of Au nanoparticles, thereby enhancing the photocatalytic degradation performance of the Au–MoS_2_ composite material. Regarding the key factors influencing photocatalytic activity, we believe that the adsorption of organic dye molecules on the photocatalyst surface plays a crucial role. The adsorption of organic molecules not only promotes their enrichment and activation on the photocatalyst surface but also affects the transfer and transport of photoinduced charges, thereby profoundly influencing the photocatalytic efficiency. Therefore, we focused on the adsorption behavior on the photocatalyst surface and explore its intrinsic relationship with photocatalytic activity. Through this work, we aim to reveal in depth the effects of Au modification and adsorption behavior in Au–MoS_2_ NCPs photocatalysts on their photocatalytic performance. This research provides theoretical guidance and experimental evidence for the development of efficient visible light-driven photocatalysts.

## 2 Experimental Section

**Figure 1:**
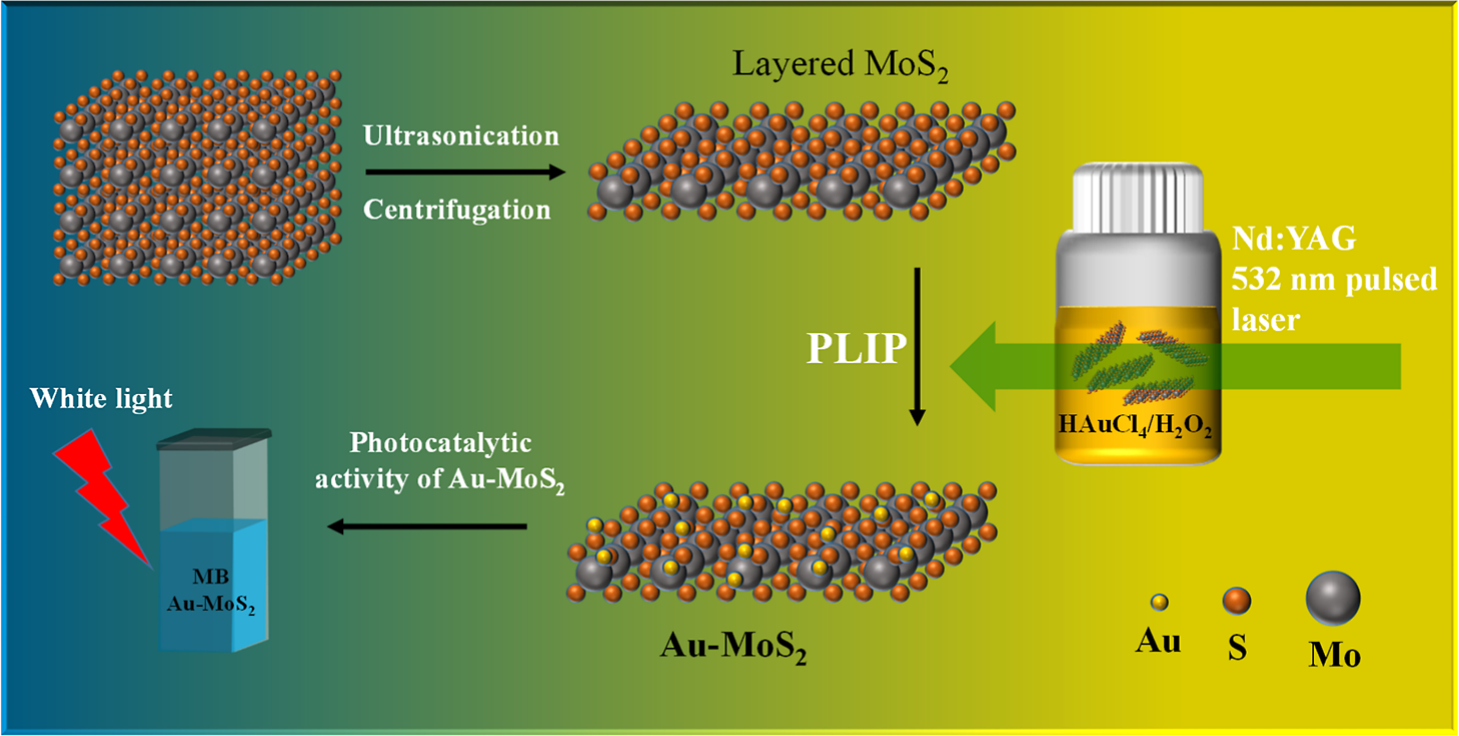
Schematic illustration of the synthesis of plasmonic Au–MoS_2_ NCP photocatalysts and their photocatalytic applications. (Upper left) Exfoliation of layered MoS_2_ into nanosheets by ultrasonication and centrifugation. (Center) Au nanoparticles uniformly decorated on the surface of MoS_2_ nanosheets via the PLIP process using a gold precursor solution. (Right) Depiction of the PLIP process involving a 532 nm pulsed laser for the in situ growth of Au NPs in the presence of H_2_O_2_ and HAuCl_4_. The resultant plasmonic Au–MoS_2_ hybrids exhibit exceptional light absorption and charge separation capabilities, enabling their application in photocatalytic degradation of organic dyes, MB.

Layered MoS_2_ was prepared using a sonication-assisted exfoliation method from bulk MoS_2_ crystals in an aqueous surfactant solution, following a procedure adapted from previous studies.^43^ Initially, 250 mg of MoS_2_ powder and 75 mg of sodium cholate (Aldrich) were added to a 50 mL aqueous solution. The mixture was subjected to ultrasonic crushing in an ice–water bath. Ultrasonication was conducted for 3 h at 50% power, followed by an additional 2 h at 70% power to achieve a black–green MoS_2_ nanosheet dispersion. Subsequent to ultrasonication, the dispersion was centrifuged at 3000 rpm for 30 min to separate the green supernatant containing the exfoliated MoS_2_ nanosheets from the bulk MoS_2_. The supernatant was further centrifuged at 12,000 rpm for 30 min to isolate the layered MoS_2_. To eliminate sodium cholate adsorbed on the nanosheet surfaces, the isolated MoS_2_ was dispersed in ultrapure water with sonication assistance. The dispersion was then centrifuged at 12,000 rpm for 30 min, and the sediment was collected to complete the washing process. This washing procedure was repeated twice more to ensure thorough removal of sodium cholate. Finally, the sediments were dispersed in a specific quantity of ultrapure water to prepare a uniform dispersion of layered MoS_2_. To ensure high reproducibility for optical absorption detection, the stock solution of layered MoS_2_ was utilized after sonication treatment for 2 min. The experimental procedure for preparing Au–MoS_2_ NCPs is as follows. First, a nanosecond pulsed laser was employed to induce the photolysis reaction of auric acid (HAuCl_4_) in an aqueous solution in the presence of a reducing agent (H_2_O_2_), with layered MoS_2_ prepared by a sonication-assisted exfoliation method. The specific reaction mechanism for the formation of Au NPs has been described in other studies.^44^ To fabricate Au–MoS_2_ NCPs, 1 mL of MoS_2_ at a concentration of 0.1 mg/mL was added to the auric acid/H_2_O_2_ precursors containing various concentrations of HAuCl_4_·3H_2_O (1 mL fixed volume), as well as a fixed concentration (2 mM) and volume (1 mL) of H_2_O_2_. The solution was then irradiated for 10 min with a pulsed Q-switch Nd:YAG laser (LS-2137U; LOTIS TII, Minsk, Belarus) with a wavelength of 532 nm, a pulse duration of 6–7 ns, a pulse repetition rate of 5 Hz, and a fluence of approximately 50 mJ/cm^2^. The laser beam was directed at the middle of the precursor solution to ensure uniform light exposure. This method primarily involves the addition of H_2_O_2_ precursor into auric acid and triggering of a photolysis reaction through the energy of intense pulsed laser light. In the photocatalytic degradation experiment of MB, a fixed volume of Au–MoS_2_ composite material (0.5 mL) and a fixed concentration (10^–5^ M) and volume (1.5 mL) of MB were placed in a standard cuvette. The absorption spectra of the cuvette under white light illumination (Thorlabs, OSL1) and time durations were recorded. The photocatalytic degradation efficiency was calculated by dividing the remaining MB concentration (*C*) at each time point by the initial MB concentration (*C*_0_). The sample was positioned 20 cm from the fiber guided light source. The electrochemical measurements, CV, were carried out on a wireless potentiostat (Zensor, ECWP100) that was connected to a screen printed electrode where the added 5 μL Au–MoS_2_ solution as working electrode, conducting carbon as the counter electrode, and Ag as the pseudo reference electrode. Figure 1 shows in detail the synthesis process and photocatalytic application of the Au–MoS_2_ NCP photocatalyst. The as purchased MoS_2_ powders are first subjected to ultrasonication and centrifugation to produce exfoliated MoS_2_ nanosheets. These nanosheets are then exposed to a PLIP process using a gold precursor solution, leading to the in situ growth of Au nanoparticles (NPs) on the surface of MoS_2_, thereby yielding the Au–MoS_2_ NCPs. A ball-and-stick model illustrates the structure of the Au–MoS_2_ hybrid, showing uniform distribution of Au NPs on the layered MoS_2_ matrix. The growth of Au NPs on the layered MoS_2_ is facilitated by a 532 nm pulsed laser in a photolysis process involving H_2_O_2_ and HAuCl_4_.

## 3 Results and Discussion

**Figure 2:**
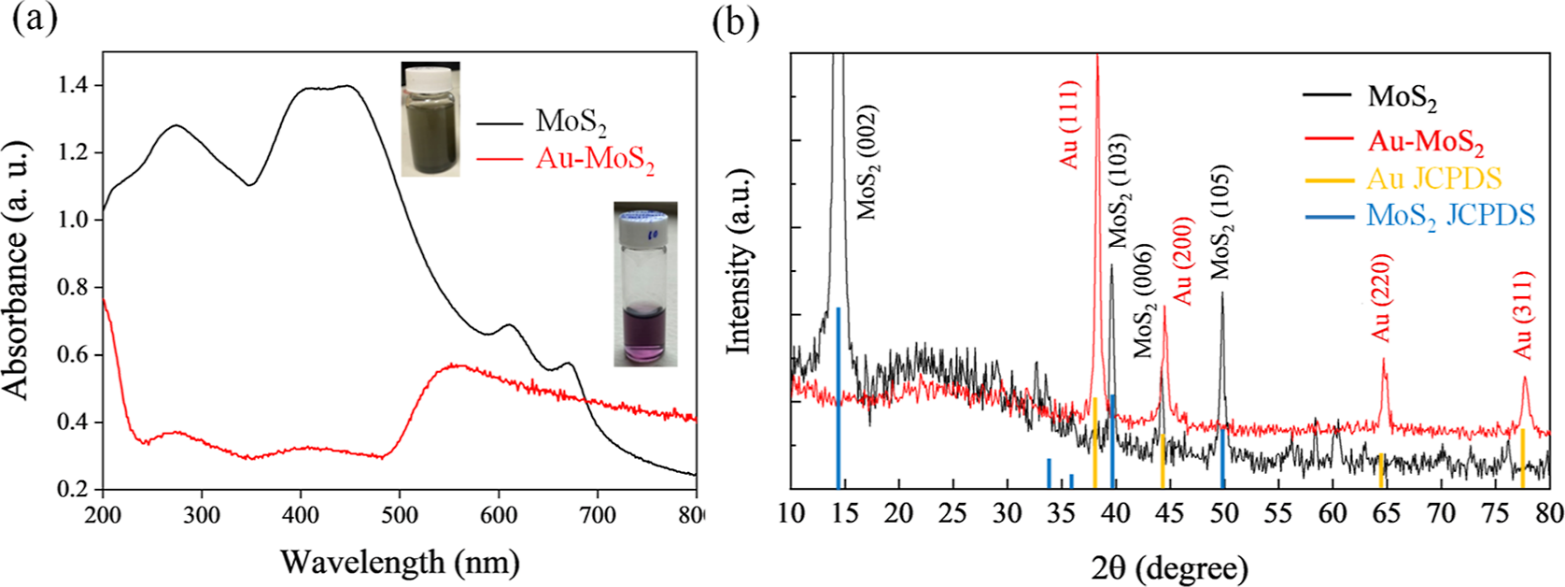
(a) UV–vis spectra of sonication–exfoliated MoS_2_ (black) showing A1 and B1 excitonic peaks, and Au–MoS_2_ NCPs (red) exhibiting a broad plasmonic band centered at 550 nm. (b) XRD patterns of sonication–exfoliated MoS_2_ (black) with peaks corresponding to 2H phase, and Au–MoS_2_ (red) with additional Au diffraction peaks at 38.2 and 44.4°. The slight protrusion at 44.2° in Au–MoS_2_ is assigned to residual ordered MoS_2_ (006) planes.

The absorption spectrum is one of the most crucial properties of layered MoS_2_. As a 2D material, the absorption spectrum of MoS_2_ is closely related to its layered structure and electronic configuration. The visible light absorption of layered MoS_2_ primarily originates from its band structure, which comprises both direct and indirect bandgaps. For monolayer MoS_2_, the direct bandgap is approximately 1.8–1.9 eV, while the indirect bandgap is around 1.2–1.3 eV.^45^ These bandgaps give rise to distinct absorption characteristics in the visible range, which are of significant importance for applications such as photocatalysis. When illuminated, MoS_2_ absorbs light, leading to the excitation of electrons from the VB to the CB, forming electron–hole pairs. Within the band structure of MoS_2_, there exist different states, denoted as A1 and B1, representing distinct bandgap transitions associated with specific energy level changes during the electron excitation process.^46^ The absorption of light at particular wavelengths corresponds to electron transitions to these different bandgap states, resulting in characteristic absorption peaks in the spectrum. Absorption spectroscopy can provide strong evidence for the significant differences in optical and electronic properties between MoS_2_ and Au–MoS_2_ NCPs. Figure 2a displays the absorption spectra of the fabricated MoS_2_ using the sonication-assisted exfoliation method and the PLIP-fabricated Au–MoS_2_ NCPs over the wavelength range of 200–800 nm. The black curve represents the layered MoS_2_ sample, exhibiting distinct A1 and B1 absorption peaks at 610 and 670 nm, respectively. According to quantum confinement theory, these two characteristic peaks correspond to the direct and indirect bandgap transitions in monolayer MoS_2_.^45^ The presence of these peaks confirms the existence of MoS_2_ nanosheets with different layer numbers in the prepared sample. Beyond 700 nm in the near-infrared region, the absorption of MoS_2_ rapidly decreases. In contrast, the absorption spectrum of the Au–MoS_2_ (red curve) undergoes a significant change. The original A1 and B1 characteristic peaks of MoS_2_ disappear completely, being replaced by a broad and intense absorption band with a maximum at 550 nm. The emergence of this absorption band is closely associated with the LSPR absorption of the Au NPs in the visible region.^47^ The PLIP method successfully incorporates Au nanoparticles into the MoS_2_ matrix, endowing the hybrid with new optical properties. The difference of optical property between layered MoS_2_ and Au–MoS_2_ NCP may be attributed to the delocalized nature of the Mo–S bonds in the rock salt structure leads to a reorganization of the electron cloud in MoS_2_, resulting in a redistribution of the electronic energy levels.^48^ Additionally, the local electric field effect alters the distribution of electronic states within MoS_2_. The combined influence of these microscopic effects reshapes the band structure of MoS_2_, eliminating its original A1 and B1 electronic transition levels. Instead, a new absorption band arises due to the LSPR effect. This remarkable change in the absorption behavior directly reflects the reshaping of electronic processes within the Au–MoS_2_ nanoheterostructure.

Figure 2b presents the XRD patterns of the fabricated MoS_2_ using the sonication-assisted exfoliation method and the PLIP-fabricated Au–MoS_2_ NCPs. The black XRD pattern of fabricated MoS_2_ exhibits characteristic peaks at around 2θ values of 14.4, 39.6, 44.2, and 49.8°, corresponding to the (002), (103), (006), and (105) planes, respectively. The positions and relative intensities of these diffraction peaks align well with the standard card (JCPDS no. 37-1492) of the 2H–MoS_2_ hexagonal phase, confirming the successful preparation of 2H–MoS_2_.^49^ Notably, the intense (002) peak arises from the layered structure of MoS_2_, where the (002) planes are exposed on the surface. For the Au–MoS_2_ NCP (red XRD pattern in Figure 2b), in addition to the characteristic peaks of MoS_2_, two distinct diffraction peaks emerge at 38.2 and 44.4°, assigned to the (111) and (200) planes of the face-centered cubic phase of Au (JCPDS no. 04-0784),^50^ verifying the successful incorporation of Au nanoparticles into the MoS_2_ matrix via the PLIP method. Compared to as fabricated MoS_2_, the Au–MoS_2_ sample exhibits a significant decrease in the intensity of the (002) peak, possibly due to the intercalation of Au NPs between the MoS_2_ layers during the PLIP process, disrupting the long-range stacking order along the *c*-axis.^51^ Additionally, the presence of Au NPs on the surface of MoS_2_ sheets could lead to X-ray absorption or scattering effects, affecting the relative intensities of MoS_2_ peaks.^43^ The laser irradiation during PLIP might have also caused localized structural changes or defects in MoS_2_, further influencing its diffraction pattern.^38^ Interestingly, a slight protrusion is observed at around 44.2° on the shoulder of the 44.4° Au peak in the Au–MoS_2_ sample, which coincides with the (006) plane of layered MoS_2_. This suggests that despite the potential disruption of the layered stacking by Au incorporation, a small fraction of MoS_2_ nanosheets may have retained some degree of ordered stacking. The weak (006) peak could arise due to the coupling interaction between Au NPs and MoS_2_, potentially inducing partial rearrangement and preferential orientation of some MoS_2_ layers.^52^

**Figure 3:**
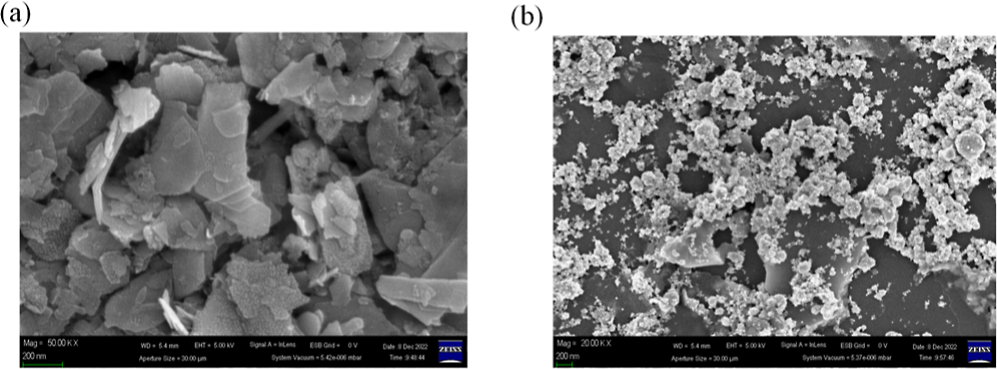
SEM images of (a) sonication–exfoliated MoS_2_ showing stacked and agglomerated nanosheet morphology, and (b) Au–MoS_2_ NCPs exhibiting well-dispersed MoS2 nanosheets decorated with Au nanoparticles of 20–50 nm diameter.

Figure 3 presents the surface morphologies of sonication–exfoliated MoS_2_ and Au–MoS_2_ NCPs investigated by SEM Figure 3a reveals that the sonication–exfoliated MoS_2_ sample comprises a large quantity of stacked nanosheet structures, exhibiting the unique 2D layered morphology characteristic of MoS_2_. This morphology endows MoS_2_ with a high specific surface area, beneficial for charge separation and transport of photogenerated carriers. However, severe agglomeration and restacking of the MoS_2_ nanosheets are observed, likely induced by interlayer van der Waals interactions, which may reduce the accessible effective surface area to some extent. In contrast, the Au–MoS_2_ nanocomposite material in Figure 3b displays distinctly different morphological features. First, numerous small metallic nanoparticles with diameters ranging from 20 to 50 nm are observed decorating the surface of the MoS_2_ nanosheet matrix. Second, compared to the pristine MoS_2_ sample, the MoS_2_ nanosheets in the Au–MoS_2_ NCPs exhibit a certain degree of exfoliation and delamination, resulting in a more dispersed and separated nanosheet morphology with increased exposure of edge and basal plane surfaces. This deagglomeration effect is likely attributed to the incorporation of Au NPs, which disrupts the interlayer van der Waals interactions in MoS_2_, facilitating its exfoliation. Such morphological changes are highly beneficial for enhancing the photocatalytic performance of the Au–MoS_2_ NCPs. On one hand, the presence of Au nanoparticles imparts the material with excellent visible light absorption and electron trapping capabilities.^47^ On the other hand, the exfoliated and dispersed MoS_2_ nanosheets provide more efficient pathways for charge transport and separation of photogenerated carriers.^45^ These complementary effects synergistically promote the overall photocatalytic reaction. Therefore, the successful incorporation and surface decoration of Au NPs onto MoS_2_ nanosheets via the PLIP method not only endows the composite with unique optical properties but also optimizes its morphological structure, favoring enhanced photocatalytic activity. These findings will provide strong support for subsequent investigations of visible light photocatalytic performance and mechanistic analyses.^53^

**Figure 4:**
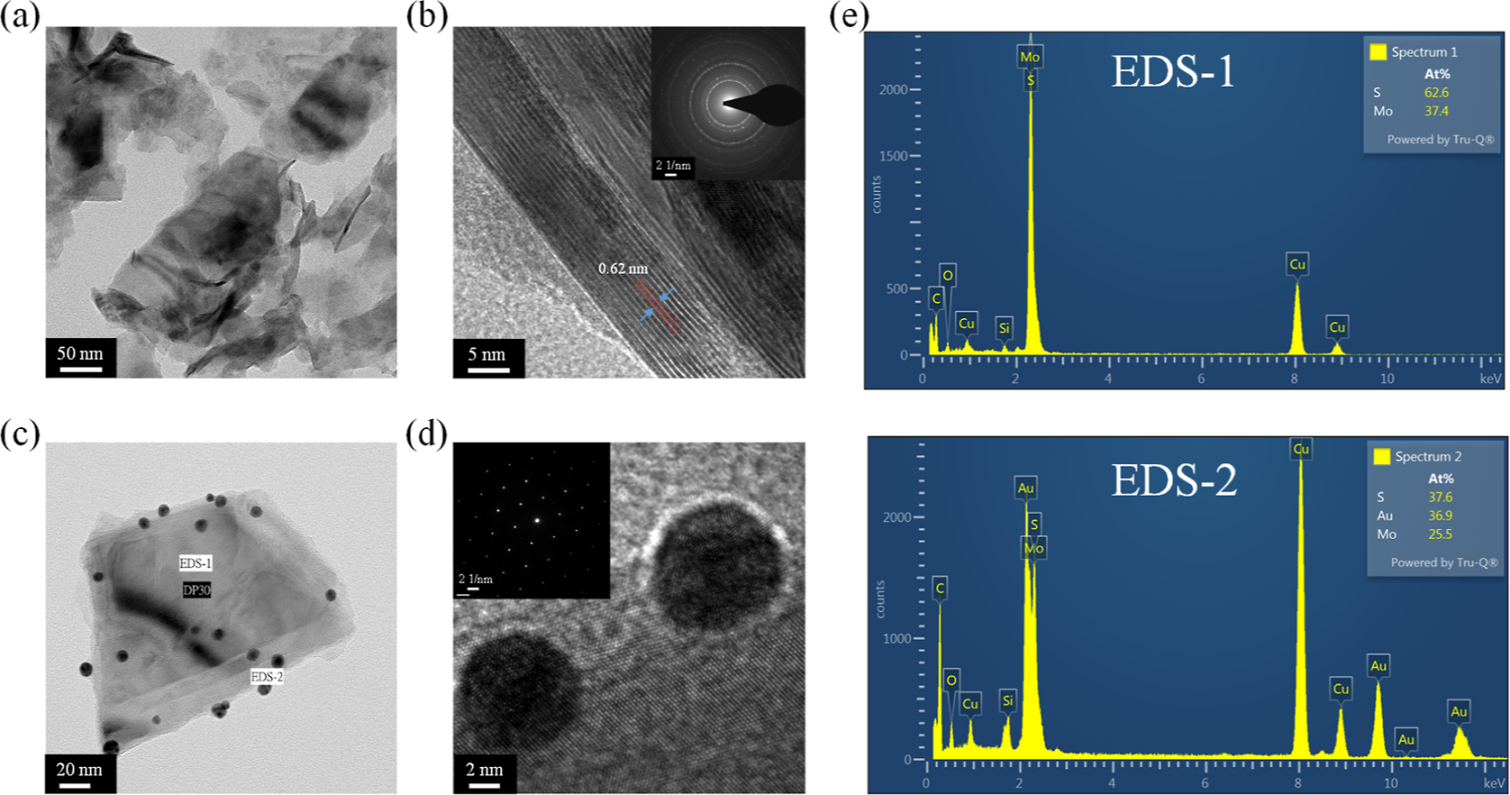
(a) TEM image of sonication–exfoliated MoS_2_ showing stacked nanosheet structure. (b) HRTEM image highlighting the atomic configuration and 0.62 nm interlayer spacing of MoS_2_. (c) TEM image of Au–MoS_2_ NCPs with Au NPs distributed on MoS_2_ nanosheets. (d) HRTEM image of Au–MoS_2_ exhibiting Au nanoparticles anchored at MoS_2_ edges and interlayers. (e) TEM–EDS elemental mapping confirming the nanoscale distribution and coupling of Au and MoS_2_.

TEM and high-resolution TEM (HRTEM) analyses provide direct insights into the nanostructural features of sonication–exfoliated MoS_2_ and the Au–MoS_2_ NCPs. The low-magnification TEM image in Figure 4a reveals that the sonication–exfoliated MoS_2_ sample comprises stacked nanosheet structures, consistent with the SEM observations. The high-resolution HRTEM image in Figure 4b further illustrates the atomic arrangement within the layered MoS_2_ structure, where each alternating bright and dark rectangular region represents the sandwich-like “S–Mo–S” configuration of a single MoS_2_ layer. Meticulous analysis yields an interlayer spacing of approximately 0.62 nm, closely matching the standard value of 0.615 nm for the 2H–MoS_2_ hexagonal phase,^49^ reaffirming the typical 2H phase structure. For the Au–MoS_2_ NCPs, the low-magnification TEM image in Figure 4c clearly displays few Au NPs uniformly distributed on the surface of the MoS_2_ nanosheets. The HRTEM image in Figure 4d reveals that Au NPs are anchored to the edges and interlayer regions of the MoS_2_ nanosheets, exhibiting intimate contact favorable for rapid charge transfer. Furthermore, the TEM–EDS elemental mapping in Figure 4e corroborates the precise distribution and coupling of Au and Mo elements at the nanoscale, providing direct evidence of the successful formation of the Au–MoS_2_ NCPs. Notably, the incorporation of Au NPs induces a certain degree of exfoliation and tilting of the MoS_2_ nanosheets, as observed in Figure 4d, exposing more edge and basal plane surfaces. This morphological change significantly increases the effective surface area, facilitating charge separation and transport, thereby enhancing the photocatalytic efficiency. In conjunction with the XRD results, the microscopic analyses provide a comprehensive understanding of the structural features of the composite photocatalyst. As previously discussed, the XRD patterns confirm the existence of the Au–MoS_2_ NCP structure through characteristic peak positions and intensities. Concurrently, the weakening of the MoS_2_(002) peak and the emergence of a faint protrusion near the Au(200) peak suggest that the incorporation of Au nanoparticles via the PLIP method induces structural distortions and reorganization within the layered MoS_2_ matrix. Such structural changes not only increase the surface area but may also modulate the band structure and active sites of MoS_2_ through mechanisms like quantum confinement effects, collectively promoting the overall photocatalytic performance.

**Figure 5:**
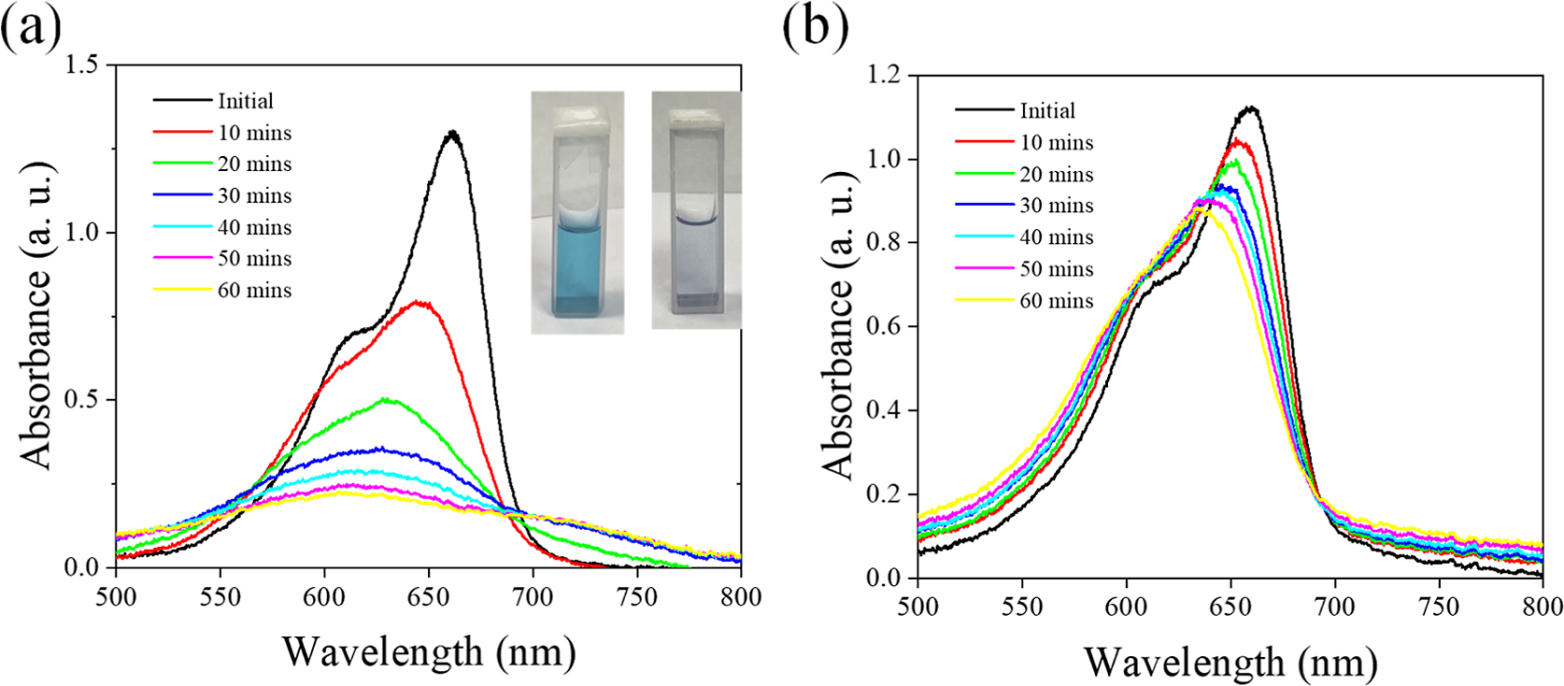
(a) Photocatalytic degradation of MB by Au–MoS_2_ under visible light, showing gradual absorbance decrease and a blue shift in the MB absorption peak. (b) Absorbance decay of MB/Au–MoS_2_ solution in the dark, attributed to MB adsorption onto the Au–MoS_2_ nanosheets.

Figure 5a shows the photocatalytic degradation behavior of MB solution under visible light irradiation, using the Au–MoS_2_ NCPs as the photocatalyst. The absorbance of the MB solution gradually decreases with prolonged irradiation time, indicating the significant visible light photocatalytic activity of Au–MoS_2_ for effectively degrading MB molecules. Interestingly, a blue shift in the MB absorption peak is observed during the photocatalytic degradation process with Au–MoS_2_. This phenomenon suggests that MB may undergo an initial step where its conjugated aromatic ring structure is disrupted, potentially generating linear intermediates such as polyketone-like species.^54−56^ These intermediates, with reduced conjugation, would exhibit a blue-shifted absorption maximum. Subsequently, these intermediates are further oxidized and ring-opened, eventually leading to complete mineralization into small molecules like CO_2_ and H_2_O. Therefore, the blue shift in the MB absorption peak likely reflects the presence of degradation intermediates. Furthermore, when MB molecules undergo electrostatic or chemical adsorption interactions with the Au–MoS_2_ nanosheet surface, the specific surface forces may influence their molecular orbitals and electron cloud densities, resulting in a shift of the absorption maximum.^54^ This molecular–surface interaction is analogous to the effect of MB interacting with other ions or molecules in solution. Generally, MB exists predominantly in an aggregated state in solution, whereas it tends to adsorb as individual molecules on the catalyst surface. The absorption maxima of the aggregated and monomeric forms of MB can exhibit a blue or red shift due to differences in intra- and intermolecular conjugation effects. The observed blue-shifted peak may reflect the transition of MB from an aggregated to a monomeric state upon adsorption.

Moreover, as shown in Figure 5b, a gradual decrease in absorbance is observed for the Au–MoS_2_/MB solution system even in the absence of light irradiation. This phenomenon is likely attributed to the adsorption of MB molecules onto the Au–MoS_2_ nanosheets, which often precedes and induces the subsequent photocatalytic reaction. Indeed, as a cationic dye molecule, MB is readily attracted to the negatively charged MoS_2_ surface through electrostatic interactions, facilitating chemical adsorption.^56^ The observed blue shift in the MB absorption peak further corroborates that upon electrostatic or chemical adsorption onto the Au–MoS_2_ nanosheet surface, MB molecules may experience changes in their molecular orbitals and electron cloud densities due to the specific surface forces exerted by the catalyst, leading to a shift or blue shift in their absorption maximum.^54^ This molecular–surface interaction is fundamentally similar to the effect observed when MB interacts with other ions or molecules in solution. As a negatively charged semiconductor material, MoS_2_ can readily attract the positively charged MB cations through electrostatic forces, potentially inducing a rearrangement of the electronic states and energy level structure of MB during the adsorption process, ultimately resulting in changes in its absorption spectrum.

**Figure 6:**
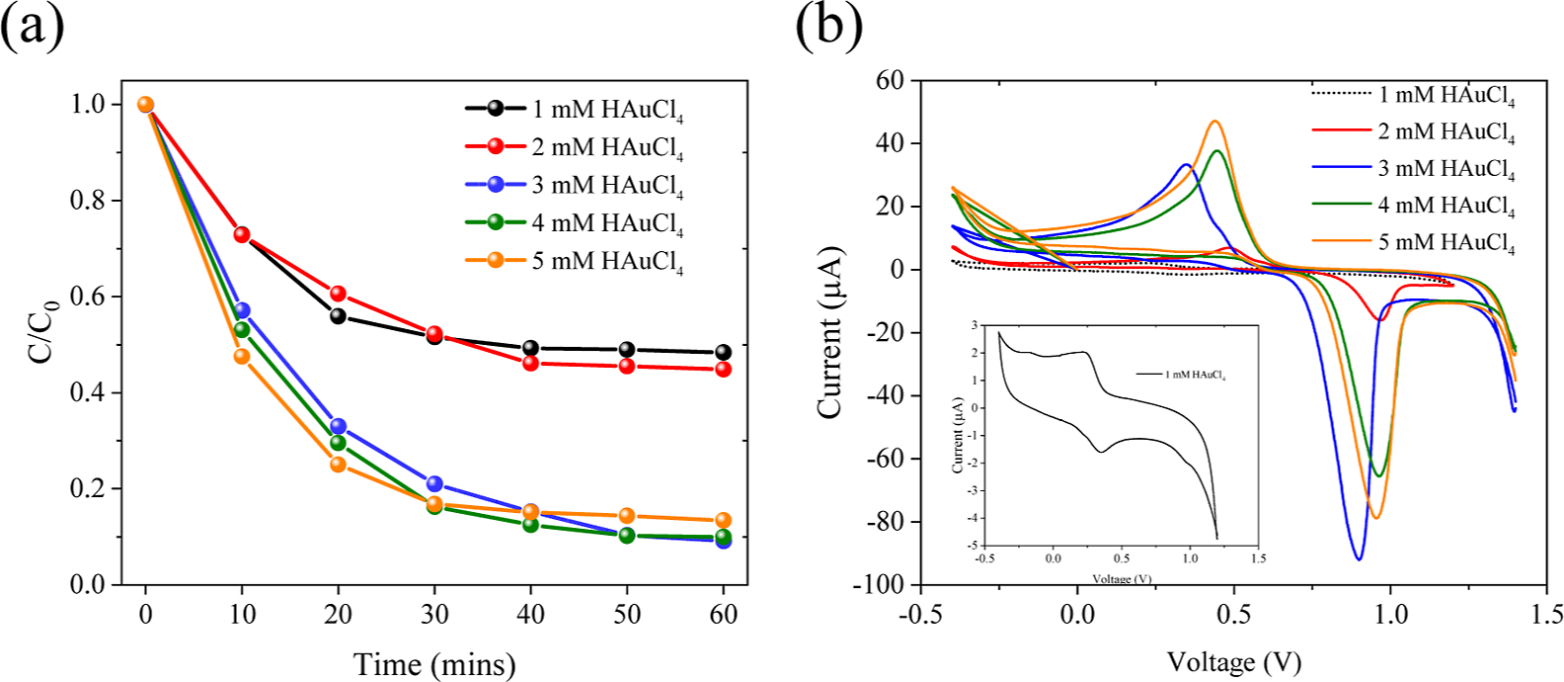
(a) Effect of Au loading on MB photodegradation over Au–MoS_2_ under visible light. Au-3 (3 mM Au) shows optimal ∼70% degradation in 20 min. Au-5 (5 mM Au) exhibits highest ∼80% degradation. Lower Au loading (Au-1, 1 mM) leads to much lower activity. (b) CV curves of Au–MoS_2_ with varying Au loadings. Higher Au content enhances redox peak intensities, indicating improved charge transfer for photocatalysis. Distinct redox peaks at high Au loadings, less pronounced peaks at low loadings.

Figure 6a further elucidates the influence of Au NPs loading on the photocatalytic degradation efficiency of MB over the Au–MoS_2_ NCPs. As the concentration of the Au precursor solution (HAuCl_4_) increases, the catalytic activity of the resulting Au–MoS_2_ samples gradually improves. The sample prepared using a 3 mM gold precursor (Au-3) exhibits optimal catalytic performance, achieving nearly 70% degradation of MB within 20 min of visible light irradiation. The Au-5 sample, synthesized with a 5 mM gold precursor, displays the highest photocatalytic activity, degrading approximately 80% of MB within the same time frame. In contrast, a lower gold precursor concentration (1 mM, Au-1 sample) results in a significantly reduced catalytic activity of the Au–MoS_2_ NCPs. These results indicate that a higher Au nanoparticle loading is more favorable for realizing the maximum photocatalytic potential of the Au–MoS_2_ hybrid material. An increased quantity of Au NPs not only enhances visible light absorption through LSPR effects but also provides additional pathways for photogenerated charge separation and transfer, thereby significantly boosting the overall photocatalytic activity. The above observations are further corroborated by electrochemical CV analyses, as shown in Figure 6b. With increasing gold precursor concentration, the Au–MoS_2_ samples exhibit more pronounced redox peak pairs in the CV curves, accompanied by a gradual increase in peak current intensities. This implies that a greater number of Au NPs are involved in the charge transfer processes, facilitating the photocatalytic reactions. Conversely, at lower Au loadings, the characteristic CV peaks become less distinct, indicating a reduced efficiency in charge carrier transfer. Collectively, these findings demonstrate that the PLIP synthesis method allows for precise control over the Au nanoparticle loading within the Au–MoS_2_ NSPs. An appropriate increase in Au content is beneficial for achieving optimal charge separation and transfer efficiencies, thereby maximizing the visible light photocatalytic activity. However, it is crucial to note that excessively high Au loadings beyond a certain threshold may lead to detrimental effects, such as nanoparticle aggregation, which could hinder the photocatalytic reactions. Therefore, maintaining an optimal Au content range is essential.

**Figure 7:**
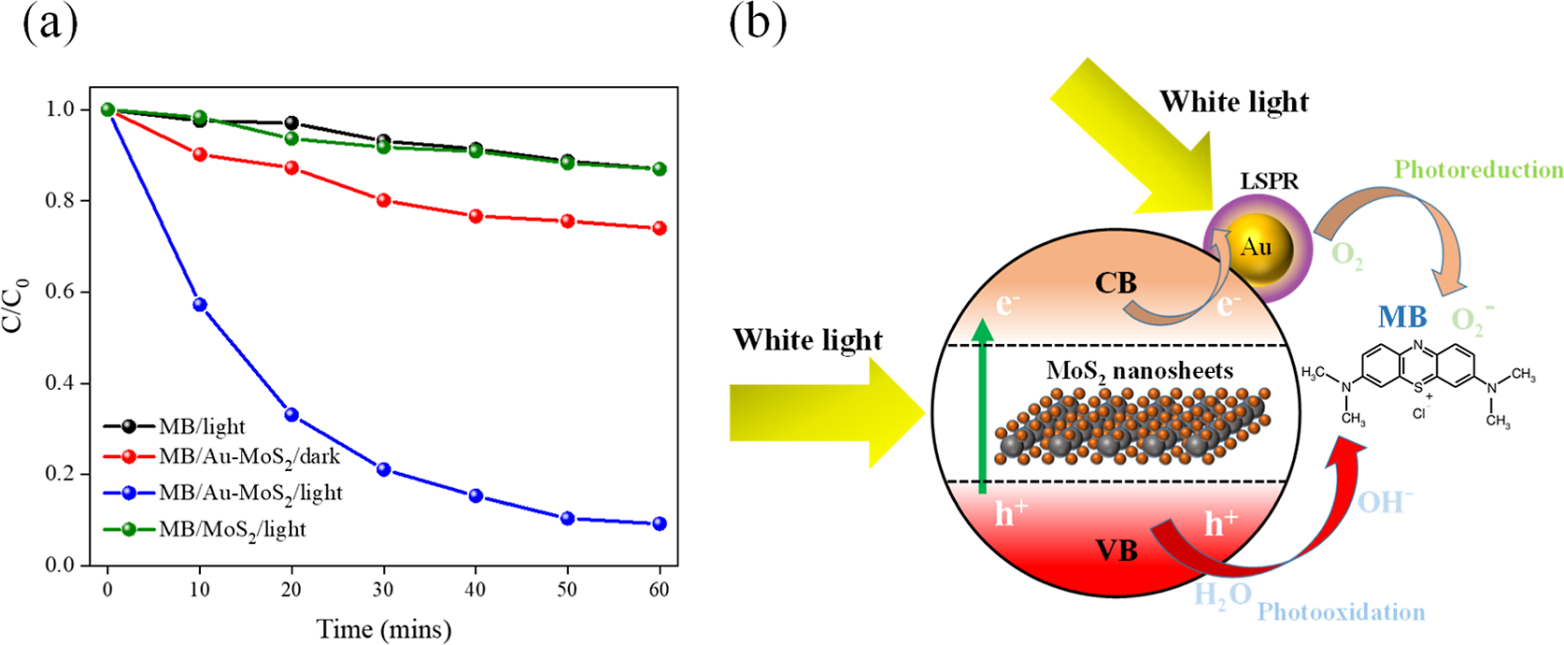
(a) Photocatalytic degradation profiles of MB under different conditions. MB/light (black) shows no degradation of MB without a catalyst under light irradiation. MB/Au–MoS_2_/dark (red) exhibits adsorption of MB onto the Au–MoS_2_ NCPs. MB/Au–MoS_2_/light (blue) demonstrates efficient photocatalytic degradation of MB over the Au–MoS_2_ NCPs under white light irradiation. MB/MoS_2_/light (green) shows no absorption or photocatalytic degradation of MB with sonication–exfoliated MoS_2_. (b) Proposed mechanism for photocatalytic degradation of MB over the Au–MoS_2_ NCPs under white light irradiation. Au NPs facilitate light absorption and charge separation via LSPR, while MoS_2_ nanosheets act as the semiconductor. Photogenerated electrons reduce O_2_ to superoxide radicals (O_2_^•–^), and holes oxidize H_2_O/OH^–^ to hydroxyl radicals (^•^OH), enabling MB degradation.

As shown in Figure 7a, the absorption spectrum of MB alone under light irradiation exhibits no photodegradation. Similarly, the photocatalytic degradation efficiency of MB over sonication–exfoliated MoS_2_ under visible light irradiation is negligible, and no significant adsorption is observed in the absence of light, unlike the case of Au–MoS_2_. The lack of photocatalytic activity toward MB over sonication–exfoliated MoS_2_ under visible light illumination is likely due to the absence of effective visible light absorption and catalytic active sites. This adsorption and activation effect can be attributed to the localized electric field enhancement induced by the Au NPs, enhancing the adsorption and activation of organic molecules on the MoS_2_ surface. Consequently, the Au-modified MoS_2_ exhibits improved visible-light photocatalytic activity toward MB degradation, and the adsorption of MB is more pronounced compared to pristine MoS_2_. It is noteworthy that in the absence of light irradiation, the concentration of MB in the Au–MoS_2_/MB mixed solution slowly decreases, which can be ascribed to the adsorption of MB molecules on the Au–MoS_2_ surface, a common precursor step for photocatalytic reactions. Compared to sonication–exfoliated MoS_2_, the Au-modified MoS_2_ NCP demonstrates superior visible-light photocatalytic activity for MB degradation. This highlights that the introduction of Au nanoparticles not only endows the material with excellent visible light responsivity but also potentially promotes the visible-light photocatalytic reaction through synergistic mechanisms, thereby enhancing the overall catalytic efficiency. The schematic, as shown in Figure 7b, illustrates the photocatalytic degradation mechanism of MB dye over Au–MoS_2_ NCPs under visible light irradiation. Upon white light illumination, the LSPR effect in the Au NPs is excited, leading to the generation of hot electrons that can be injected into the CB of the MoS_2_ nanosheets. The injected electrons in the MoS_2_ CB participate in photoreduction reactions with adsorbed oxygen molecules, forming superoxide radical anions (O_2_^•–^). On the other hand, the holes in the VB of MoS_2_ can oxidize water or hydroxide ions to produce highly reactive hydroxyl radicals (OH^–^). These ROS, including O_2_^•–^ and OH^–^, are responsible for the degradation of the adsorbed MB dye molecules through oxidation reactions. Notably, the adsorption of positively charged MB molecules on the negatively charged MoS_2_ surface is facilitated by electrostatic attractions, ensuring close proximity between the dye and the catalytically active sites. This adsorption process plays a crucial role in enhancing the photocatalytic degradation efficiency by enabling efficient charge transfer and ROS-mediated oxidation reactions at the interface. In addition to the plasmonic effects of Au NPs, the formation of a Schottky barrier at the Au–MoS_2_ interface may also contribute significantly to the enhanced photocatalytic activity of the Au–MoS_2_ NCPs.^54^ Considering the small band gap of MoS_2_, the LSPR effect of Au NPs may have a limited contribution to the overall photoactivity. Instead, the Schottky barrier formed between Au and MoS_2_ could play a key role in the efficient separation and transfer of photogenerated charge carriers. The Schottky barrier arises from the band alignment between the Au NPs and the MoS_2_ nanosheets, creating an internal electric field at the interface. This electric field promotes the directional migration of photogenerated electrons from MoS_2_ to Au, while the holes remain in the MoS_2_ VB. The spatial separation of electrons and holes effectively suppresses their recombination, leading to an enhanced photocatalytic activity. Moreover, the Schottky barrier can also lower the activation energy for charge transfer across the Au–MoS_2_ interface, further facilitating the photocatalytic reactions.

## 4 Conclusions

The results demonstrate the successful synthesis of Au–MoS_2_ heterogeneous NCP photocatalysts via the PLIP method and elucidate their efficient visible-light-driven mechanism for the degradation of MB dye. The incorporation of an optimal amount of Au NPs significantly enhances the photocatalytic activity of MoS_2_ through several synergistic pathways. First, the plasmonic Au NPs improve the visible light harvesting capability of the composite. Second, hot electron injection from the Au NPs promotes charge separation within the heterojunction. Third, the Au/MoS_2_ nanoscale interface facilitates favorable charge transfer between the two components. Furthermore, the electrostatic adsorption of cationic MB molecules onto the negatively charged Au–MoS_2_ surface enhances interfacial reactions, contributing to the overall photocatalytic efficiency. These complementary effects synergistically boost the visible-light photocatalytic performance of the Au–MoS_2_ NPs toward efficient MB degradation. The photogenerated electrons and holes participate in the generation of superoxide and hydroxyl radicals, respectively, subsequently initiating the oxidative degradation of MB. Therefore, rational design of the Au–MoS_2_ heterojunction structure, coupled with tuning the Au content and charge state of MoS_2_, offers a promising strategy for developing highly efficient visible-light-driven nanocomposite photocatalytic systems with potential applications in organic pollutant remediation and energy catalysis. The results provide valuable insights for developing efficient visible-light-driven metal–semiconductor composite photocatalysts, suggesting that tuning the noble metal component loading can optimize charge separation and transfer pathways, thereby maximizing photocatalytic activity. The complementary characterization techniques provide mutually corroborating evidence, unambiguously demonstrating the efficient integration of Au NPs with MoS_2_ nanosheets via the PLIP method. The rational design and optimization of Au–MoS_2_ heterojunctions, coupled with tuning the Au content and charge state of MoS_2_, offer a promising strategy for developing highly efficient visible-light-driven nanocomposite photocatalytic systems. These Au–MoS_2_ NCPs hold immense potential for applications in organic pollutant remediation, water splitting for hydrogen production, CO_2_ reduction, and other energy conversion processes driven by visible light irradiation. The insights gained from this study pave the way for the design and development of advanced nanocomposite photocatalysts for addressing environmental and energy challenges.
